# Tertiary Intratumor Lymphoid Tissue in Colo-Rectal Cancer

**DOI:** 10.3390/cancers4010001

**Published:** 2011-12-28

**Authors:** Francesca Bergomas, Fabio Grizzi, Andrea Doni, Samantha Pesce, Luigi Laghi, Paola Allavena, Alberto Mantovani, Federica Marchesi

**Affiliations:** 1 Department of Immunology and Inflammation, IRCCS Humanitas Clinical Institute, Via Manzoni 56, 20089 Rozzano, Milan, Italy; E-Mails: francesca.bergomas@humanitasresearch.it (F.B.); andrea.doni@humanitasresearch.it (A.D.); samantha.pesce@humanitasresearch.it (S.P.); paola.allavena@humanitasresearch.it (P.A.); alberto.mantovani@humanitasresearch.it (A.M.); 2 Laboratory of Molecular Gastroenterology, IRCCS Humanitas Clinical Institute, Via Manzoni 56, 20089 Rozzano, Milan, Italy; E-Mails: fabio.grizzi@humanitasresearch.it (F.G.); luigi.laghi@humanitas.it (L.L.); 3 Department of Gastroenterology, IRCCS Humanitas Clinical Institute, Via Manzoni 56, 20089 Rozzano, Milan, Italy; 4 Department of Translational Medicine, University of Milan, Milan 20089, Italy

**Keywords:** colon cancer, immune infiltration, ectopic lymphoid tissue

## Abstract

Ectopic (or tertiary) lymphoid tissue develops at sites of inflammation or infection in non lymphoid organs and is associated with chronic inflammation. In colon mucosa, small lymphoid aggregates are already present in homeostatic conditions, as part of the gut-associated lymphoid tissue and play an essential role in the immune response to perturbations of the mucosal microenvironment. Despite the recognized role of inflammation in tumor progression, the presence and biological function of lymphoid tissue in cancer has been poorly investigated. We identified aggregates of lymphocytes resembling tertiary lymphoid tissue in human colorectal cancer specimens; intratumor accumulations of lymphocytes display a high degree of compartmentalization, with B and T cells, mature dendritic cells and a network of CD21^+^ follicular dendritic cells (FDC). We analyzed the adaptation of colon lymphoid tissue in a murine model of colitis-associated cancer (AOM/DSS). B cell follicle formation increases in the context of the chronic inflammation associated to intestinal neoplasia, in this model. A network of lymphatic and haematic vessels surrounding B cell follicles is present and includes high endothelial venules (HEV). Future task is to determine whether lymphoid tissue contributes to the persistence of the tumor-associated inflammatory reaction, rather than represent a functional immune compartment, potentially participating to the anti tumor response.

## 1. Introduction

Immune responses can develop independently of secondary lymphoid organs, in tertiary lymphoid tissue (or organs), which develops ectopically at sites of inflammation or infection in peripheral, non-lymphoid organs [1,2,3]. In these pathologic settings, tissues harboring target antigens are infiltrated by cellular effectors of the immune system, which organize anatomically and functionally as in secondary lymphoid organs (lymph nodes, spleen), with formation of B-cell follicles and T-cell areas. The possibility that these organized lymphoid aggregates behave as functional immune sites for T lymphocyte activation and B cell maturation and antibody production is still debated.

The process of lymphoid neogenesis has been observed in several chronic inflammatory conditions [4,5], in autoimmune diseases [6,7,8], infectious diseases [9], and chronic graft rejection [10]. The relevance of ectopic follicle formation to the disease relies on the functional capability to sustain an immune response; in autoimmune conditions it would sustain local production of autoantibodies, as it has been shown in rheumatoid arthritis [8,11]. On the other hand, the development of ectopic lymphoid tissues may help eradicating pathogens and infectious agents. As a matter of fact, ectopic aggregates sustain an *in situ* immune response and the priming of T cells against inhaled antigens in an animal infection model with formation of bronchus-associated lymphoid tissue [12].

Infiltration by immune cells of growing human solid tumors is well documented in a wide variety of distinct tumor types, however, few reports exist that ectopic lymphoid tissue is present also in solid tumors.

Despite accumulating evidence indicates that the immune system is a critical determinant of tumor outgrowth, its role is paradoxical, depending on the cellular and molecular mediators involved in the shaping of the tumor microenvironment. The role of cells of the innate immunity, including macrophages, neutrophils and myeloid derived suppressor cells in promoting cancer progression is recognized in a wide variety of tumor types [13]. Lately, also B cells have been shown to be crucial in establishing the chronic inflammation associated with *de novo* carcinogenesis [14]. The mechanism is likely to be antibody-dependent, with the deposition of IgG immune complexes, activation of innate immune cells, including tumor associated macrophages (TAM), through the FcγR [14,15] and polarization to an immunosuppressive phenotype. In contrast, a high number of T lymphocytes is an indicator of good prognosis in different tumor types, (including melanoma) [16] as well as ovarian [17] and colon cancer [18,19], suggesting that an antitumor T-cell immune response may take place *in vivo* in patients with solid tumors.

B cell aggregates containing CD21^+^ follicular DCs have been identified as ectopic lymphoid follicles in breast cancer [20,21] and NSCLC [22]. Their precise functions and association with tumor progression, however, is poorly characterized. A functional ectopic lymphoid tissue may burst the cancer-associated inflammation, being crucial site for antibody production, with important effects on macrophage and myeloid cell polarization [15]. On the other hand, an organized immune response taking place in the follicles may possibly control tumor invasion and metastasis, increasing the efficiency and specificity of T-cell priming and allowing a faster T cell reaction to tumor antigens *in situ*.

The dual interplay between the immune system and cancer is very well represented in colon-rectal cancer (CRC). An immune/inflammatory infiltrate, including lymphocytes, neutrophils, and macrophages, is present in CRC and is mainly concentrated along the invasive margin (tumor-host interface). Epidemiologic and preclinical studies have confirmed that inflammatory bowel disease is a recognized risk factor for developing colon cancer [13,23], infiltrating T lymphocytes have a protective role in this tumor, being significantly associated with better clinical outcome [18,19,24]. Although there is evidence that tumor prognosis is related to the homing of effector immune cells to the tumor site, it is still unclear where the activation of a specific immune response takes place. Whether B and T lymphocytes infiltrating human colon cancer organize in lymphoid structures, potentially contributing to their immune activation is still unknown.

## 2. Results and Discussion

### 2.1. Ectopic Lymphoid Tissue is Present in the Mucosa of Colon-Cancer Patients

**Figure 1:**
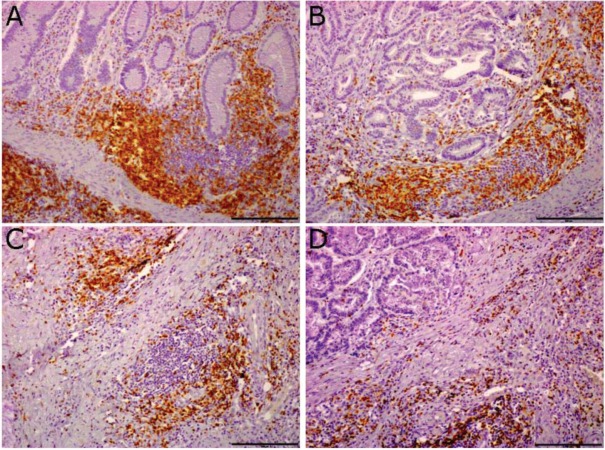
Tertiary lymphoid tissue in sections of human colo-rectal tumors. Immunohistochemistry with an anti-CD3^+^ antibody allows identification of lymphoid tissue in human colon mucosa. Lymphoid tissue is present in normal mucosa, at the bottom of the crypts (**A**) and in tumor sections, both adjacent to tumor nests (**B**) and in tumor-associated stromal tissue (**C**); CD3^+^ T lymphocytes scattered in the tissue are shown in (**D**). Scale bar = 200 μm.

The presence of intratumor infiltrating lymphocytes in the mucosa of colon cancer patients has been documented and evaluated in detail. Importantly, quantitative and qualitative analysis of CD3^+^ lymphocytes infiltrating colon cancer have revealed how they importantly associate with better prognosis in cancer patients [18,19]. We found that tumor infiltrating lymphocytes are not only scattered throughout the stroma and interspersed between tumor cells (Figure 1D); they also cluster in aggregates resembling tertiary lymphoid tissue (Figure 1B,C). Lymphoid tissue in the normal colon mucosa is usually located at the bottom of the crypts and shows a distinct compartmentalization between a T and a B cell zone (Figure 1A). In tumor sections, lymphoid tissue is found both adjacent to tumor nests (Figure 1B) and in stromal regions, at the invasive front of the tumor (Figure 1C). The presence of lymphoid aggregates in the stromal tissue associated to the tumor suggests that they may be *de novo* formed structures; their location at the tumor-host interface may have important consequences for the immune response.

**Figure 2:**
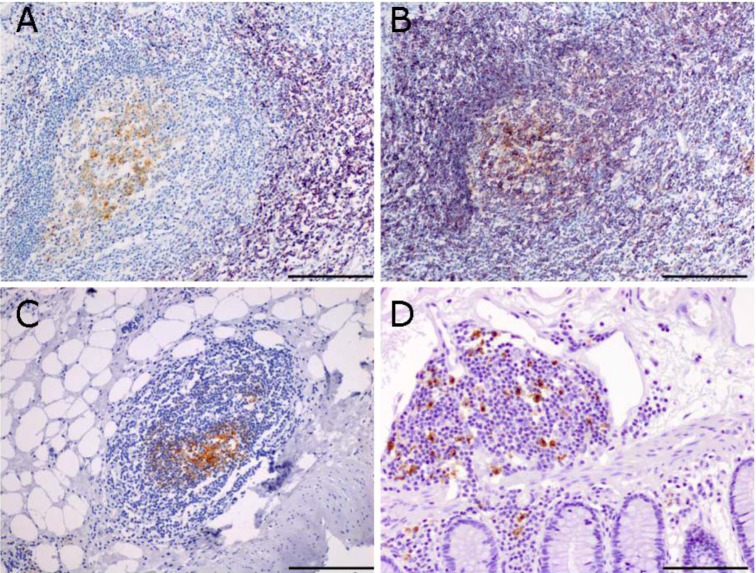
Immunohistochemistry of tertiary lymphoid tissue in sections of human colo-rectal tumors. T cells (purple, **A**) and CD20^+^ B cells (purple, **B**) surround CD21^+^ follicular dendritic cells (brown, **A–C**); Sections in A and B are consecutive sections and show the compartmentalization between B and T cells in the follicle. Mature dendritic cells are also present, as evidenced by DC-LAMP staining (**D**). Scale bar = 100 μm.

Staining with an antibody specific for CD21 identifies the presence of a network of follicular dendritic cells (FDC) inside the follicles (Figure 2A-C). The presence of FDCs is particularly significant, because they play a crucial role in affinity maturation by antigen selection of B cells with high affinity mutated antigen receptors, suggestive of an ongoing process of B cell activation inside the follicles. Antigen-driven oligoclonal expansion of tumor-infiltrating B cells has been shown in infiltrating ductal carcinoma of the breast [20]. Tumor-associated antigens sampled and processed by dendritic cells could potentially be in direct contact with specific T cells inside the follicles, thereby increasing the efficiency and specificity of the priming. Therefore, intratumor lymphoid tissue may contribute to a faster lymphocyte reaction to the shifting expression profile of tumor antigens. Mature dendritic cells are present, as evidenced by DC-LAMP immunoreactivity, and may contribute to T cell activation in the follicles. The presence of mature dendritic cells in intratumor lymphoid tissue has been associated to better prognosis in non-small cell lung cancer [22].

### 2.2. Characterization of Lymphoid Tissue in the Colon of Mice Developing Colitis-Associated Cancer

**Figure 3:**
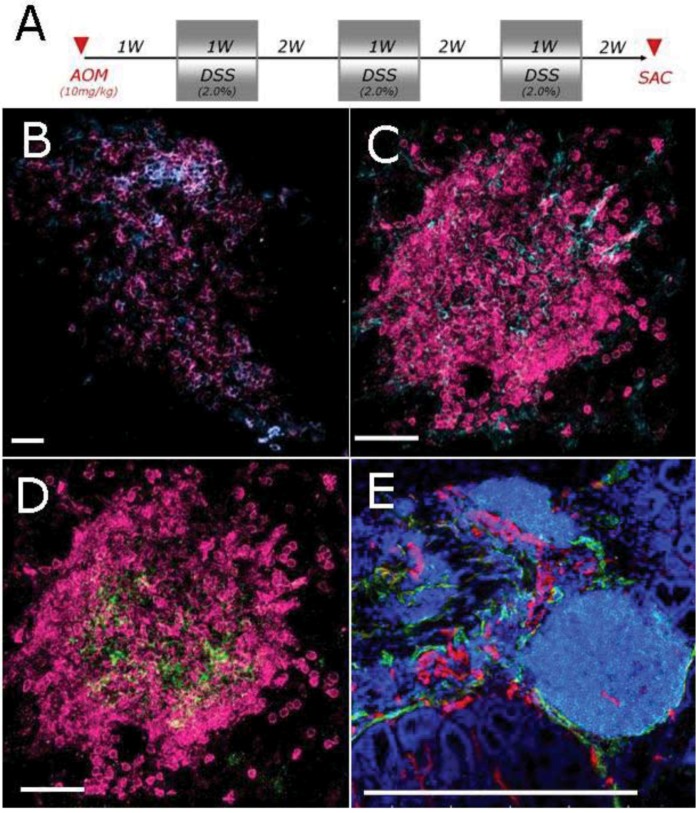
Characterization of lymphoid follicles in the colon of mice subjected to AOM/DSS. Schematic AOM/DSS administration protocol (**A**); Frozen sections of distal colons were stained with specific antibodies, including: B220/CD3 (magenta/cyano, **B**), B220/CD11c (magenta/cyano, **C**), B220/FDC (magenta/green, **D**) and B220/CD31/Lyve1 (cyano/magenta/green, **E**). B cells are the most represented cell population in the follicles. CD11c^+^ dendritic cells are also present. Lymphoid follicles are vascularized by CD31^+^ haematic vessels as well as Lyve1^+^ lymphatic vessels. Scale bar: 30 μm (**B–D**), 500 μm (**E**).

Colon cancer is the paradigm of the connection between inflammation and cancer and mechanistic studies in preclinical models have helped clarifying how inflammatory mediators play a role in the progression from colonic inflammation to cancer. We analyzed the adaptation of B cell follicles in the colonic mucosa of mice subjected to AOM/DSS protocol, which develop colitis-driven polyps in the colon, as a result of the combined treatment with the carcinogen azoxymethan (AOM) and the mucosal irritant dextran sulphate (DSS) (Figure 3A). Lymphoid follicles can be easily recognized in the colon mucosa of the mouse, as they are part of the gut associated lymphoid tissue (GALT). B cells are the most represented cell population in the colonic follicles, with few scattered T cells (Figure 3B). Similarly to lymphoid tissue in human colon sections, among dendritic cells, 2 populations are present: CD11c^+^ conventional dendritic cells and FDC^+^ follicular dendritic cells (Figure 3C,D). Lymphoid structures are also vascularized by CD31^+^ haematic vessels as well as Lyve1^+^ lymphatic vessels (Figure 3E).

Aggregates contain all the components required for antigen-driven B-cell proliferation and maturation in a germinal center, including FDCs, although the B cells are not located in a well-defined mantle zone and dark and light zones of the germinal center are not evident. The vessel network draining the follicles allows an active and continuous trafficking of cells between the follicle and the tumor tissue.

### 2.3. Expansion of Lymphoid Tissue in the Context of the Chronic Inflammation Associated to Intestinal Neoplasia

**Figure 4:**
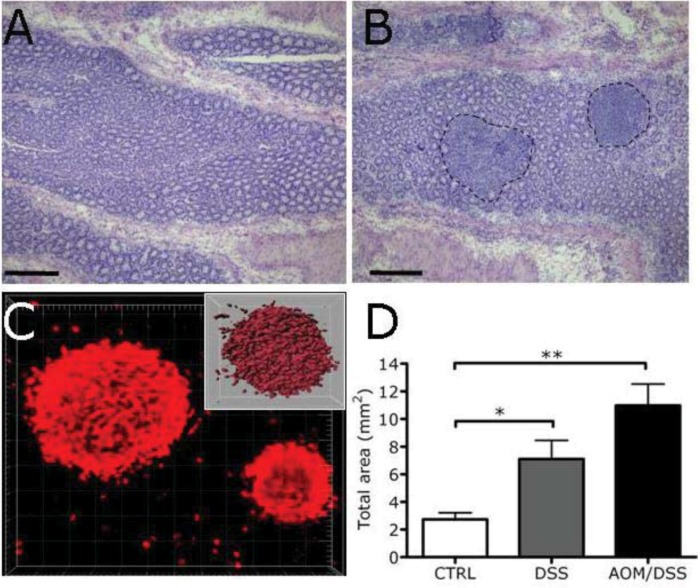
B cell follicles increase in a model of colon carcinogenesis. (**A**, **B**) Representative H&E of colon mucosa in CTRL mice and after the AOM/DSS treatment. Dot lines show lymphoid aggregates. Scale bar = 100 μm; (**C**) Morphometric analysis of the lymphoid aggregates by 3D visualization of B220^+^ follicles (in red) in colon whole mounts. Isosurfaces were obtained by importing confocal RGB image stacks to Imaris software (inset); (**D**) Quantification of volume of the lymphoid aggregates. (n = 3 mice, Ctrl; n = 3 mice DSS; n = 3 mice AOM/DSS; bars represent SEM).

Alterations of the gut microenvironment are reflected in adaptation of pre existing lymphoid tissue, which can increase in size and in number. Lymphoid follicles in the colon mucosa of the mice mostly localize in the submucosal region of the intestine and can be visualized in horizontal sections of the intestine, as dense aggregates of lymphocytes (Figure 4A,B). Mice treated with AOM/DSS and developing tumors in the colon showed an expansion of lymphoid aggregates in the colonic mucosa (Figure 4B). We performed a careful and precise quantification of the size and number of the lymphoid aggregates, by morphometric analysis of lymphoid aggregates after 3-dimensional visualization of B220^+^ follicles in colon whole mounts (Figure 4C). Quantification of B cell follicle volume showed a significant increase in the amount of lymphoid tissue, in the mice developing tumors compared to untreated mice (Figure 4D). Notably, comparison between control mice and mice treated with DSS alone (inducing colitis) showed that the expansion of follicles is already present in colitic mice, likely because of the massive disruption of the intestinal epithelial barrier and bacteria stimulation. Therefore, the chronic inflammatory condition associated to tumor development induces expansion of lymphoid follicles in this model.

## 3. Experimental Section

### 3.1. Patients

Cancer-tissue specimens were obtained from patients who underwent resective surgery for colorectal cancer at the IRCCS Istituto Clinico Humanitas, from January 1997, to November 2004. Tissues from patients who underwent neoadjuvant radiotherapy for rectal cancer or with perioperatively detected metastases were excluded.

### 3.2. Immunohistochemistry

Formalin-fixed, paraffin-embedded, and 2-μm thin sections of tumour were deparaffinised and exposed to an antigen-retrieval system for 30 min, before being incubated with the specific antibody for 1 hour at room temperature. Antibodies used were mouse anti-human CD3 (clone F7.2.38, Dako), mouse anti-human CD20 (clone L26, Dako), rabbit anti-human CD21 (clone EP3093, Abcam) and rat anti-human DC-LAMP (clone 1010E1.01, Dendritics). Endogenous peroxidase was blocked with 3% hydrogen peroxide for 20 min at room temperature. Reactive sites were identified by exposure to a secondary antibody (HRP rabbit/mouse; Dako Envision) for 30 min at room temperature and finally counterstained with haematoxylin.

### 3.3. Immunofluorescence

Frozen sections of colons were stained with the following specific antibodies: anti-mouse B220 (clone RA3-6B2, e-bioscience) anti-mouse CD3 (clone 145-2C11, e-bioscience), anti-mouse CD11c (clone N418, e-bioscience), anti-mouse Lyve1 (rabbit polyclonal, Abcam), anti-mouse CD31 (clone 2H8, Millipore), anti-mouse FDC (clone FDC-M1 BD, Pharmingen). The three-dimensional visualization of B cell follicles was obtained on colon whole mounts; briefly, colons were removed from mice and immersed in 1% paraformaldehyde fixative solution for 2 hours. Tissues were then washed and stained immunohistochemically by incubating whole mounts with primary antibodies diluted in PBS containing 0.3% Triton X-100, 2% bovine serum albumin, 5% normal goat serum, 0.01% glicine and 0.1% sodium azide overnight, followed by overnight incubation with secondary antibodies.

### 3.4. Morphometric Analysis

Morphometric analysis of colon whole mounts was performed by 3D visualization of B220^+^ follicles. Isosurfaces were obtained by importing confocal RGB image stacks to Imaris software. For each colon analyzed (n = 3 mice each experimental group), one stack of each follicle identified was acquired and analyzed.

## 4. Conclusions

Established evidence points to a role of inflammatory cells and mediators in cancer progression and possibly in the carcinogenesis process. The importance of defining the presence of tertiary lymphoid tissue at the tumor site arises from the possibility of an organized immune response taking place in the follicles and possibly controlling tumor invasion and metastasis. In line with previous data indicating the relevance of intratumor lymphocytes in colon cancer, we find that the presence of lymphoid structures in the tumor context deserves attention. We show evidence that ectopic lymphoid tissue in the mucosa of colon cancer patients display a very organized structure, with distinct T cell and B cell zone, suggesting the possibility to sustain an efficient immune response. On the other hand, in a model of colitis-associated cancer, in which inflammation drives polyp formation, the expansion of lymphoid tissue suggests that it may sustain the inflammatory reaction or contribute to the suppression of the antitumor immune response. Future task is to determine whether tertiary lymphoid tissue contributes to the persistence of the tumor-associated inflammatory reaction, rather than represent functional immune structures, actively participating to the anti tumor response. Preclinical models can help defining their function and the molecular mechanisms involved in their formation.
